# Effect of Photoperiod on Chinese Kale (*Brassica alboglabra*) Sprouts Under White or Combined Red and Blue Light

**DOI:** 10.3389/fpls.2020.589746

**Published:** 2021-01-11

**Authors:** Jiaxuan Chen, Zeyuan Chen, Zunwen Li, Yijiao Zhao, Xiaodong Chen, Gefu Wang-Pruski, Rongfang Guo

**Affiliations:** ^1^Joint FAFU-Dalhousie Lab, College of Horticulture, Fujian Agriculture and Forestry University, Fuzhou, China; ^2^Institute of Horticultural Biotechnology, College of Horticulture, Fujian Agriculture and Forestry University, Fuzhou, China; ^3^Department of Plant, Food, and Environmental Sciences, Faculty of Agriculture, Dalhousie University, Truro, NS, Canada

**Keywords:** photoperiod, glucosinolate, myrosinase, Chinese kale, red/blue lights

## Abstract

To determine the response of Chinese kale (*Brassica alboglabra*) sprouts to photoperiods under different light sources, we used four photoperiods (0-h light/24-h dark, 8-h light/16-h dark, 12-h light/12-h dark, and 16-h light/8-h dark) to investigate their sprout growth and secondary metabolite glucosinolates (GSs) accumulation under white or combined red-and-blue (RB) light sources. We found that the 16-h light condition under RB light produced plants with the greatest dry matter. Sprouts grown under 16-h RB light condition achieved greater length than those under white light. To investigate the role of RB light in plant growth and GS accumulation, we applied RB light sources with different RB ratios (0:10, 2:8, 5:5, 8:2, and 10:0) to cultivate sprouts. The results showed that significant differential accumulation of GSs existed between sprouts grown under blue (RB, 0:10) and red (RB, 10:0) light; there was greater GS content under blue light. The underlying mechanism of differential GS content in sprouts under red or blue light condition was studied using RNA sequencing technique. Interestingly, abundant GS biosynthetic gene transcripts were observed in sprouts grown under red light compared with under blue light. The expression of β-glucosidase family homolog genes related to GS degradation differed under red and blue light conditions, among those *TGG4* homolog was detected with higher expression under red light than with blue light. Taking into consideration, the lower GS accumulation in sprouts under red rather than blue light, we conclude that the degradation of GSs may play a key role in sprouts GS homeostasis.

## Introduction

Chinese kale sprouts are widely regarded as a healthy vegetable through their substantial nutritional components and strong antioxidant ability. Originating in China, Chinese kale sprouts contain abundant glucosinolates (GSs), vitamin C, and polyphenols. Practices such as adding sugar, applying different light, adding NaCl, and using hormone treatments have been used to promote the quality of kale sprout production (Guo et al., 2011, 2013; Qian et al., 2016).

GS biosynthesis can be regulated by treatment with light of different wavelengths. The effect of light quality on GS accumulation differs by species. For example, blue light (470 nm) exposure has been found to accelerate aliphatic GS levels, while decreased indolic GS accumulation in *Cardamine fauriei* (Abe et al., 2015). Application of blue light at 450 or 470 nm increased the total GS content in turnips (Antonious et al., 1996) and broccoli (Kopsell and Sams, 2013), respectively, whereas red light (650 nm) treatment increased the accumulation of aliphatic GSs in watercress (*Nasturtium officinale*) (Engelen-Eigles et al., 2006).

The GS biosynthetic pathway mainly includes three steps: extension of side chains, synthesis of core structures, and secondary modification of the side chains. Those steps involved multiple enzymes, such as branched-chain amino aminotransferase (BCAT), methylthioalkylmalate synthase (MAM), isopropylmalate dehydrogenase (IPMDH), the cytochrome P450 monooxygenase gene family 79s (CYP79), CYP83, and 2-oxoglutarate-dependent dioxygenase (AOP) (Kroymann et al., 2001; Sønderby et al., 2010). During chain elongation, BCAT, MAM, and IPMDH were involved. In the synthesis of core structure, both the CYP79 and CYP83 families have substrate specificity, in which CYP79F1 catalyzes the methionine to aliphatic aldoxime. The aldoximes are converted to an aci-nitro compound by CYP83A1 and then formed S-alkyl-thiohydroximate, which is cleaved by a C-S lyase (SUR1) and glucosyltransferase (UGT74B1) into desulfoglucosinolates; that process is followed by sequential glucose and sulfate transfer by the SOT enzymes to complete the basic GS skeleton. The side-chain modification of aliphatic GSs is mainly carried out by AOP and flavin-containing monooxygenase (FMOGS-OX). The AOP2 plays key a role in the formation of alkyl GSs (Neal et al., 2010), and FMOGS-OX is responsible for catalyzing the first step of modification–sulfur oxidation (Kong et al., 2016). CYP81F and indolic GS methyltransferase (IGMT) are essential in the process of indolic GSs. *CYP81F* family genes conduct hydroxylation reactions of the GS indolic ring, leading to the formation of 4-hydroxy-indol-3-yl-methyl and 1-hydroxy-indol-3-yl-methyl GSs from indolyl-3-methyl GSs, and those hydroxy intermediates are converted to 4-methoxy-indol-3-yl-methyl and 1-methoxy-indol-3-yl-methyl GSs by IGMT1 or IGMT2. During the transition of day to night, the expression of genes related to GS metabolism was changed, and the GS accumulation was affected in *Arabidopsis*; e.g., treatment with light enhanced the expression of *BCAT4*, *MAM1*, and *AOP2*, whereas the expression of GS transcription factors MYB28, MYB29, and MYB76 was reduced after transfer to darkness for 44 h (Huseby et al., 2013).

In addition to the regulation of GS biosynthesis by light, the accumulation of GSs is also affected by its own degradation. Upon tissue damage, the degradation of GSs in plants has been well studied, and in that regard, myrosinase plays an important role (Wittstock and Burow, 2010). Myrosinase belongs to the β-glucosidase (BGLU) family (Morant et al., 2008). Thus far, 47 genes encoding BGLU have been identified in the *Arabidopsis* genome (Xu et al., 2004); among them, *BGLU34*–*BGLU39* have been the most studied and named thioglucoside glucohydrolase (TGG). However, the mechanism of GS degradation in undamaged tissues is still in its infancy. The fluctuation of GSs in the transition of development stages (Brown et al., 2003) and the coordination of GS metabolism under limited nitrogen and sulfur condition (Jeschke et al., 2019) indicate the degradation of GSs occurs in intact living tissues. Until now, two atypical myrosinase PEN2/BGLU26 and PYK10/BGLU23 were demonstrated to function in the indole GS turnover in intact plants (Clay et al., 2009; Nakano et al., 2017). Recently, in the review of Sugiyama and Hirai, BGLU28 and BGLU30 were proposed to be possibly related to the GS breakdown in sulfur deficiency and darkness condition (Sugiyama and Hirai, 2019). Myrosinase activity can be regulated by multiple environmental factors (Bhat and Vyas, 2019). Both ascorbic acid (Burmeister et al., 2000) and substrate concentration (Shikita et al., 1999) could affect the myrosinase activity. Biotic stress including herbivore feeding (Shikita et al., 1999) and aphid attack (Kuśnierczyk et al., 2007), as well as abiotic stress such as salt addition (Guo et al., 2013), plant hormone treatment (Guo et al., 2014; Yan et al., 2014), drought (Koh et al., 2015), and heat (Cocetta et al., 2018), would change the transcripts of myrosinase gene or alter the abundance of myrosinase protein (Shikita et al., 1999; Burmeister et al., 2000). Illumination with blue light abruptly up-regulated mRNA levels of myrosinase and its activity (Yamada et al., 2003). High light intensity (300 μmol/m^2^/s) can also increase myrosinase activity (Charron and Sams, 2004). Prolonged darkness increased the myrosinase activity in *Arabidopsis* (Brandt et al., 2018).

Plants depend on light receptors, such as cryptochromes (CRYs) and phytochromes (PHYs), to sense light signals for day–night transitions, photoperiods, and light quality for growth and development (Lepisto and Rintamaki, 2012). After perception, complex light signaling networks induce morphogenetic and photoperiodic development by removing the photomorphogenic repressors of constitutive photomorphogenic 1 (COP1) and PHY-interacting bHLH (basic helix–loop–helix) factors (PIFs) and subsequently accumulating the positive transcription factors, including long hypocotyl 5 (HY5) and long hypocotyl in far-red 1 (HFR1) (Bae and Choi, 2008). The dynamic of GSs during the day and night has been reported in *Arabidopsis* seedlings (21-day-old) (Huseby et al., 2013). GS biosynthetic genes could be induced by light, and the GS content is low under extended darkness (Huseby et al., 2013). Sprouts refer to young seedlings 2–9 days after germination (Guo et al., 2016). It is interesting to know whether the GS pathway in sprouts would be affected by light changes. In the present study, the morphogenesis and GS accumulation were studied in Chinese kale sprouts under different photoperiod conditions. Two sources of light were provided by two panels: one was for white (W) light, and the other was for combined red-and-blue (RB) light. After selecting a suitable photoperiod, different ratios of RB light (RB, 10:0; RB, 8:2; RB, 5:5; RB, 2:8; and RB, 0:10) were used to cultivate the sprouts for better appearance and rich GS accumulation. The differential accumulation of GSs in the sprouts under the combined RB light at 10:0 and 0:10 was noted, and the corresponding molecular mechanism was investigated using RNA sequencing.

## Materials and Methods

### Plant Material and Cultivation Conditions

Seeds of Chinese kale (*Brassica alboglabra* cv. HuangHua) were purchased from Gaoda seed company (Fuzhou, China). Before the experiments, broken, deformed, and rot seeds were removed. A layer of perlite was made in culture dishes, and distilled water was added to infiltrate the perlite to place the seeds. Seeds were covered and incubated at 28°C for 1 day and then transferred to another incubator (25°C). The artificial light sources came from two light panels: one gave W light, and the other gave RB light at different ratios (RB, 10:0; RB, 8:2; RB, 5:5; RB, 2:8; and RB, 0:10). The wavelength used in this study is 640 nm for red light and 460 nm for blue light. The light-emitting diode (LED) array was 130-cm length and 50-cm width. The photoperiod was set to 0-h light/24-h dark, 8-h light/16-h dark, 12-h light/12-h dark, and 16-h light/8-h dark. The light intensity was measured by a spectrum analyzer (HiPoint, HR-350) and adjusted to 150 μmol/m^2^/s by a controller. The Chinese kale sprouts were collected for measurement of parameters including plant height, cotyledon width, fresh weight, and dry weight after growing in different light treatments for 2, 3, 6, and 9 days. The phenotypic analysis was repeated four times, and each time, four replicates were used. During each replicate, the growth parameters of four representative sprouts were recorded. The sprout samples were rapidly and gently collected from the culture dishes and washed with ddH_2_O and then were frozen in liquid nitrogen immediately and kept in polyethylene bags at −80°C for RNA extraction and GS analysis. For GS content analysis, sprouts under different treatments were collected, and four biological replicates were performed for each treatment. For RNA extraction and sequencing analysis, three biological replicates were conducted for blue- and red-light treatments, respectively.

### Estimation of GS Content in Chinese Kale Sprouts

Glucosinolates were extracted and analyzed as previously described (Guo et al., 2019) with minor modifications. Sprouts (200 mg) were boiled in 2 mL ddH_2_O for 10 min. After transferring the supernatant to a new tube, the residues were boiled with another 2 mL ddH_2_O. Then a DEAE A25 Sephadex (Sigma, A25120) (35 mg) column (pyridine acetate form) was used to let the combined aqueous extract go through. The column was washed with 500 mM pyridine acetate and ddH_2_O. The 0.1% sulfatase (Sigma, S9626) was added for overnight and eluted twice with ddH_2_O, which was desulfated GSs. Ortho-nitrophenyl-β-d-galactopyranoside (oNPG, Sigma N1127, St. Louis, MO, United States) was used as an internal standard for the high-performance liquid chromatography (HPLC) analysis and added to the sample before measurement. HPLC analysis was performed using an HPLC system consisting of a Waters 2695 separations module and a Waters 2996 photodiode array detector (Waters Corp., Milford, MA, United States). The HPLC system was connected to a computer with Empower Pro software. The sample went through a Hypersil C18 column (5 μm particle size, 4.6 × 250 mm; Elite Analytical Instruments Co., Ltd., Dalian, China) in a 30°C oven at a flow rate of 1.0 mL/min. The procedure of GS detection was 1.5% acetonitrile and 98.5% ddH_2_O (0–5 min; isocratic), 20% acetonitrile and 80% ddH_2_O (5–20 min; linear), 20% acetonitrile and 80% ddH_2_O (25–35 min; isocratic), and 1.5% acetonitrile and 98.5% ddH_2_O (35–40 min; isocratic). A 20-μL sample was injected, and the absorbance was detected at 226 nm. The individual GS content was calculated using oNPG and the response factors of desulfo-GS to oNPG (Cai et al., 2016). The measurements were performed in four biological replicates, and each biological replicate contains four experimental replicates. Four samples containing 10 to 15 sprouts in each treatment were used to perform the analysis of GS content and profiles.

### RNA Extraction, Library Construction, and RNA-Seq

Total RNA of Chinese kale sprouts was extracted using RNAiso Plus kit (Takara, 9109) from RB at the ratio of 0:10 groups (HHB) and 10:0 groups (HHR) with three biological replicates in each group, respectively. Each replicate contains at least 10 seedlings for each group. The quality and quantity of RNA were controlled by the detection using NanoDrop 1000 spectrophotometer (Thermo Fisher Scientific, Wilmington, DE, United States) and Qubit^®^ 2.0 Fluorometer (Life Technologies, Carlsbad, CA, United States), respectively. The qualified RNA was enriched with polyA tail by magnetic beads with OligodT, followed by removal of rRNA with DNA probe. The mRNA was obtained after digestion with DNaseI and RNaseH and fragmented by adding an interrupting reagent under high temperature conditions, and then the double-stranded cDNA was synthesized using the interrupted mRNA as a template. The libraries were constructed followed the procedure of purification and recovery, end repair, the base “A” addition, adaptor connection, fragment size selection, and amplification. After quality test by Agilent 2100 Bioanalyzer and ABI StepOnePlus Real-time PCR System, the qualified paired-end libraries were subjected to RNA sequencing (RNA-seq) analysis (BGI sequencing, Shenzhen, China). The sequencing data have been uploaded to NCBI SRA database (PRJNA649862).

### Data Filtering and Differentially Expressed Genes Annotation

The raw reads with low quality, linker contamination, and excessively high levels of unknown base were filtered out, and the obtained clean reads were aligned to the reference genome^1^. Gene function was annotated using *Arabidopsis thaliana* based on the National Center for Biotechnology Information and Kyoto Encyclopedia of Genes and Genomes databases. Expression level of each unigene was calculated using RSEM^2^ and exhibited as fragments per kilobase per million values. The differentially expressed genes (DEGs) were detected using the DEGseq R package (Wang et al., 2010). Genes with a *Q* ≤ 0.001 and — log2 (HHR/HHB) — > 2 were screened for significantly DEGs.

### Statistical Analysis

**FIGURE 1:**
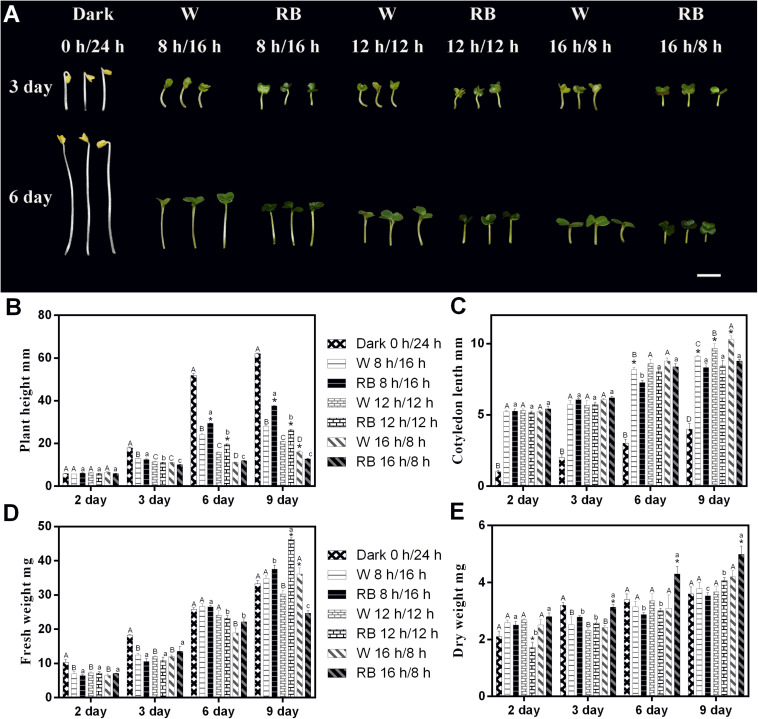
Morphology and related physiological indicators of Chinese kale sprouts under different photoperiods with W light and RB (8:2) light. **(A)** Morphology change of Chinese kale sprouts under four photoperiods (0-h light/24-h dark, 8-h light/16-h dark, 12-h light/12-h dark, and 16-h light/8-h dark) with white (W) and combined red-and-blue (RB, 8:2) light sources, respectively. Effect of these photoperiods treatments on the **(B)** plant height, **(C)** cotyledon length, **(D)** fresh weight, and **(E)** dry weight of Chinese kale sprouts. The *X* axis represents the growth days under different photoperiod conditions (2, 3, 6, and 9 days). W, white; RB, combined red and blue. The phenotype analysis was performed in four biological replicates, and four sprouts were used in each treatment. Each data point is the mean of four replicates per treatment. The capital letters (A, B, and C) mean the value is significant different under W light source and dark condition. The lower cases (a, b, and c) mean the value is significant different in RB light source. The asterisks (*) mean the value is significant different in the comparison of data in the same photoperiod under white or combined red/blue light source.

Statistical analysis was performed by using SPSS (version 19.0, Chicago, IL, United States). One-way analysis of variance and Tukey honestly significant differences multiple-comparisons test were applied to analyze the differences among different photoperiods under white light or combined red/blue light condition. The significant value was labeled with capital letter (A, B, and C) under W light source and dark condition and labeled with lower case (a, b, and c) in RB light source in Figure 1. For comparison of data in the same photoperiod under white or combined red/blue light source, the independent-samples *t*-test was used, and the significant one was labeled with asterisk (^∗^) in Figure 1. The structural formulas were drawn using Indraw^3^. Heatmaps were drawn using TBTools^4^.

## Results

### Effect of Different Photoperiods on the Morphogenesis

The phenotype of 3- and 6-day-old Chinese kale sprouts grown with different photoperiods condition under W or RB light (8:2) is shown in Figure 1A. Sprouts grown under dark conditions showed only elongation of the hypocotyls. Under the 18-h light condition, both types of sprouts grew with shorter hypocotyls and wider cotyledons irrespective of whether a W or combined RB light source was used (Figure 1A). Growth indicators (including plant height, cotyledon length, fresh weight, and dry weight) under different photoperiodic treatments were measured on days 2, 3, 6, and 9 (Figures 1B–E). Consistent with the phenotype presented in Figure 1A, the plant height and cotyledon length responded rhythmically to the illumination time. With prolonged illumination, the height decreased (Figure 1B), and the cotyledon became larger (Figure 1C). Differential responses of the plants to different light sources with the same photoperiod were noted. Under a combined RB light (8:2) source, the plant height was much greater, and the cotyledon length was significantly shorter in 9-day-old sprouts than in those grown under W light (Figures 1B,C).

Regarding the change in biomass under the four photoperiods, a difference began to emerge on day 3 with sprouts grown in the dark had the greatest fresh weight (Figure 1D). On day 6, no significant differences were evident. On day 9, sprouts grown under RB light with the 16-h light/8-h dark regimen had the lowest fresh weight (Figure 1D) but the highest dry weight (Figure 1E).

### Effect of Different Photoperiods on the GS Content

**FIGURE 2:**
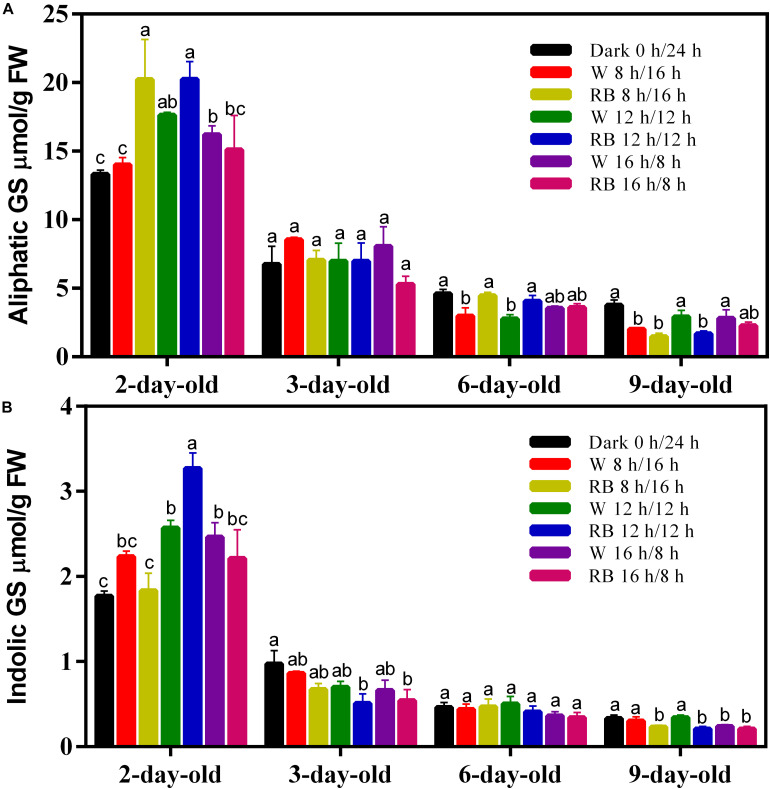
Glucosinoalte contents including **(A)** aliphatic GS and **(B)** indolic GS of sprouts grown under different photoperiods with W light or RB (8:2) light. The measurement was performed in four biological replicates and four sprouts were used in each treatment. W, white; RB, combined red and blue. Each data point is the mean of four replicates per treatment. The significant different value of GS content under different growth conditions was indicated by different lower cases.

The GS content in the sprouts at different growing stages was measured in dark (0 h/24 h), short-light (8 h/16 h), medium-light (12 h/12 h), and long-light (16 h/8 h) photoperiods under W or RB light. Aliphatic GS is the larger part in sprouts and four kinds of aliphatic GSs including glucoiberin (GIB), progoitrin (PRO), gluconapin (GNA), and glucoerucin (GER) were identified, with GNA being the most abundant one (Supplementary Table 1). Another four kinds of indolic GSs including glucobrassicin (GBS), 4-hydroxyglucobrassicin (4-OHGBS), 4-methoxyglucobrassicin (4-OMGBS), and neoglucobrassicin (NGBS) were also identified in the sprouts. The effect of photoperiod on GS content differed in sprouts at different stages. In 2-day-old sprouts, extended illumination time increased the aliphatic (Figure 2A) and indolic GS (Figure 2B) content except the short photoperiod (8 h/16 h) condition. On day 6, RB light (8:2) source gave higher aliphatic GSs to sprouts in short and medium photoperiod conditions (Figure 1A). On day 9, both the aliphatic and indolic GSs were reduced in sprouts grown under medium photoperiod (Figures 2A,B). The results showed that GS content was greatest on day 2, and it decreased gradually with the growth of the sprouts in all the tested photoperiods. Prolonged illumination and changing the light source did not affect the GS decline in the sprouts (Figures 2A,B), suggesting that reducing the GS content in sprouts may be critical to seedling development. and external factors, such as photoperiod and light source, have little effect on that process.

### Effect of RB Light at Different Ratios on the Morphogenesis

**FIGURE 3:**
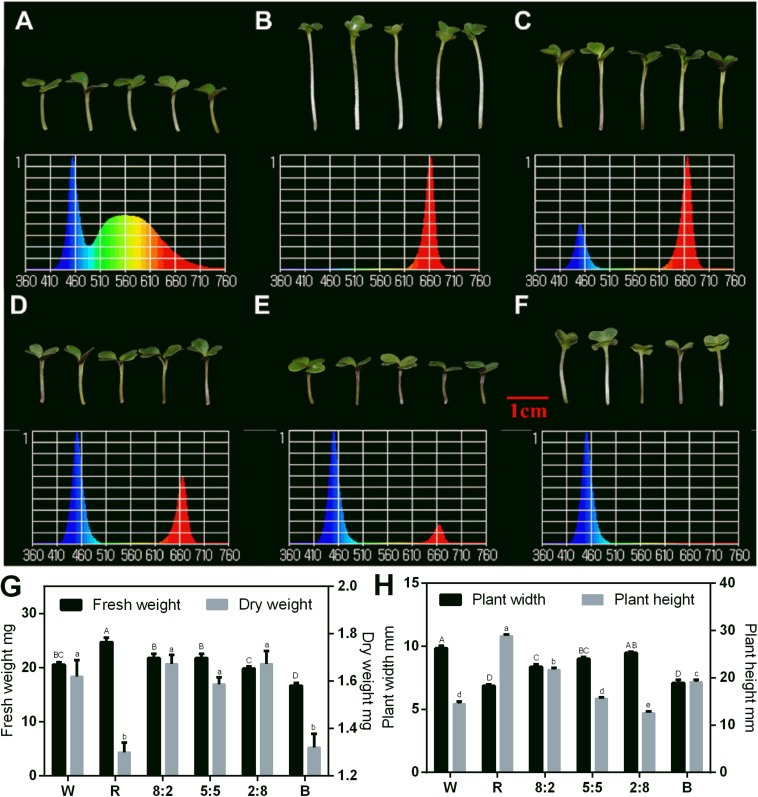
Morphology, spectral distribution, and related physiological indicators of 6-day-old Chinese kale sprouts under RB light at the 16 h-light/8 h-dark regime. Morphology of Chinese kale sprouts under **(A)** white light (abbreviated as W); **(B)** RB, 10:0 (abbreviated as R); **(C)** RB, 8:2; **(D)** RB, 5:5; **(E)** RB, 2:8; and **(F)** RB, 0:10 (abbreviated as B) conditions. Effect of different light treatments with varied RB ratios (W, R, 8:2, 5:5, 2:8, and B) on the fresh weight and dry weight **(G)** and plant width and plant height **(H)** of the sprouts. Total photosynthetic photon flux (PPF) was 150 ± 5 μmol/m^2^/s in each treatment. Spectral scans were measured at 10 cm from LED lighting sources and at center point. W, white; R, red and blue light at the ratio of 10:0; B, red and blue light at the ratio of 0:10. The phenotype analysis was performed in four biological replicates, and each biological replicate contains four samples of each treatment. Each data point is the mean of four replicates per treatment. The capital letters indicate the significant different data of fresh weight in **(G)** and plant width in **(H)**. The lower cases indicate the significant different value of dry weight in **(G)** and plant height **(H)**.

The combined RB light source had a positive impact on the sprout growth. Thus, we tested the effect of RB light at different ratios on the sprouts using W light as a control in the following study. The phenotypes and spectra under different light conditions are shown in Figures 3A–F, and the effect of RB light at different ratios on growth indicators of the sprouts was analyzed (Figures 3G,H). The fresh weight under red light condition was the greatest, and it decreased with reduction of the red-light proportion. Blue light treatment produced plants with the lowest fresh weight (Figure 3G). However, the dry matter content under blue light was as high as that with red light (Figure 3G). The sprouts’ height was greatest under red light condition, and it decreased with reduction of the red light proportion (Figure 3H). Adding blue light promoted the dwarfing of the sprouts and increased the plant width. However, the size of cotyledons of sprouts grown under single blue light was comparable with those under red light.

### Effect of Combined RB Light at Different Ratios on GS Content

**FIGURE 4:**
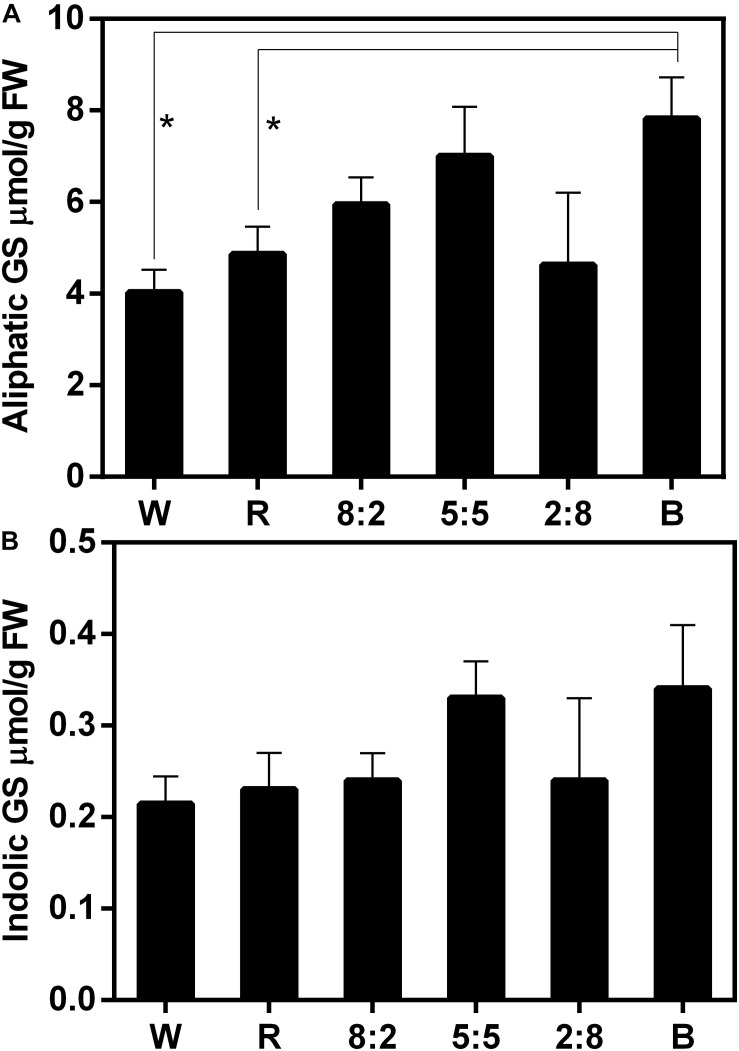
Glucosinolate content including **(A)** aliphatic GS and **(B)** indolic GS of Chinese kale sprouts under different red and blue light ratios at the 16h-light/8h-dark regime. The *X* axis represents the different treatments with varied red and blue light ratio. White (W) is the control, red (R) means RB at the ratio of 10:0, 8:2 means RB at the ratio of 8:2, 5:5 means RB at the ratio of 5:5, 2:8 means RB at the ratio of 2:8, and blue (B) means RB at the ratio of 0:10. RB means combined red and blue light. The measurement was performed in four biological replicates, and each biological replicate contains four samples of each treatment. Each data point is the mean of four replicates per treatment. The asterisks (*) indicate the significant difference in comparison of aliphatic GS content under W, R, and B conditions.

The effect of combined RB light at different ratios on GS accumulation was examined (Figure 4 and Supplementary Table 2). Compared with under W light, the aliphatic GS content in sprouts exposed to RB of 8:2, RB of 5:5, and blue light increased to varying degrees: the GS content under blue light increased significantly; treatment with red light had no effect on GS accumulation (Figure 4A and Supplementary Table 2). In addition, treatments of combined RB light at different ratios did not affect the accumulation of indolic GSs (Figure 4B and Supplementary Table 2). These results indicate that single red-light treatment cannot increase aliphatic GS content, whereas the addition of blue light can lead to such an increase in aliphatic GS content in the sprouts.

### Gene Expression Related to GS Metabolism Under RB Light

**FIGURE 5:**
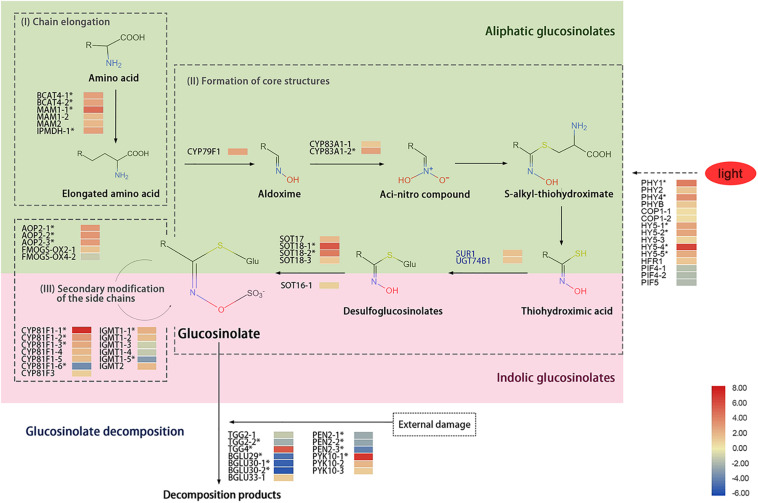
A diagram showing glucosinolate metabolism and heat map of related genes in 6-day-old sprouts under red or blue light at the 16h-light/8h-dark regime. Three biosynthetic processes including (I) chain elongation, (II) core structure formation, and (III) side-chain secondary modification were illustrated. The light green part is for the synthesis of aliphatic glucosinolate, and the pink part is for the synthesis of indolic glucosinolate. The SUR1 and UGT74B1 in blue font represent genes function in both aliphatic and indolic glucosinolate biosynthetic pathways. The solid arrows represent the glucosinolate biosynthetic pathway, the dash arrow is for unknown pathway, and the asterisks (*) represent the significantly differentially expressed genes in HHR (vs. HHB). HHR, Chinese kale sprouts under red and blue light at the ratio of 10:0; HHB, Chinese kale sprouts under red and blue light at the ratio of 0:10. The gene expression data were from libraries of HHR and HHB, each library containing three biological replicates.

The lower accumulation of GSs under red light and higher accumulation of GSs under blue light was interesting. To determine the mechanism of differential GS accumulations in sprouts under RB light, transcriptome of sprouts under RB of 10:0 (HHR) and RB of 0:10 (HHB) conditions was analyzed. The biosynthesis of GSs includes three processes: chain elongation, core structure formation, and secondary modification of the side chain (Figure 5).

During chain elongation, the precursor amino acid undergoes deamination, condensation, isomerization, and oxidative decarboxylation; it adds a methylene group (-CH2−) to the side chain of the amino acid. Two *BCAT4* gene homologs involved in deamination, two *MAM1* gene homologs and one *MAM2* gene homolog for condensation, and one *IPDMH* gene homolog for isomerization and oxidative decarboxylation were detected. Among them, expression of *BCAT4-1*, *BCAT4-2*, *MAM1-1*, and *IPMDH* homologs was significantly up-regulated by red light treatment compared with under blue light (Figure 5).

The formation of the GS core structure mainly involves the cytochrome P450 monooxygenase gene family (*CYP79* and *CYP83*) and other related genes. Ten genes including one *CYP79F1* homolog, two *CYP83A1* homologs, one *SUR1* homolog, one *UGT74B1* homolog, and five *SOT* homologs were detected in the kale sprouts in this study. Under red-light condition, the transcripts of *CYP79F1*, *CYP83A1-2*, *SOT18-1*, and *SOT18-2* homologs in sprouts were more significantly up-regulated than those under blue light (Figure 5).

The last step for GS synthesis is secondary modification of the side chains, which is responsible for the diversity of GSs. Three *AOP2* gene homologs, two *FMOGS-OX* gene homologs, seven *CYP81F* gene homologs, and six *IGMT* gene homologs were identified in the HHB and HHR libraries. Owing to the diversity of the GS side-chain modification products and their different responses to light treatments, the expression levels of those related gene homologs varied. Expression of *AOP2-1*, *AOP2-2*, and *AOP2-3*; *CYP81F1-1*, *CYP81F1-2*, *CYPF1-3*, and *CYPF1-4*; and *IGMT1-1* homologs was significantly up-regulated, whereas transcripts of *CYP81F1-6* and *IGMT1-5* homologs were significantly reduced by red light (Figure 5).

Myrosinase is the major enzyme for the turnover of GSs. Thirteen TGGs related to myrosinase were identified in the kale sprouts, including three typical myrosinase (two *TGG2* homologs and one *TGG4* homolog) and 10 atypical myrosinase enzymes (one *BGLU29* homolog, two *BGLU30* homologs, one *BGLU33* homolog, three *PEN2* homologs, and three *PYK10* homologs). Notably, expression of *TGG2*, *BGLU29-30*, and *PEN2* homologs in HHR was lower than that in HHB, whereas expression of *TGG4*, *BGLU33*, and *PYK10* homologs was significantly up-regulated in HHR (Figure 5).

### Gene Expression Related to Red or Blue Light

Four *PHY* gene homologs (*PHY1*, *PHY2*, *PHY4*, and *PHYB*) related to red light recognition were identified from the HHR and HHB libraries, and their expression levels were higher under red light (Figure 5). In addition, expression of negative regulator PIF homologs was reduced after treatment with red light. Transcription factors (including five *HY5* homologs and one *HFR1* homolog) were also detected, and among those genes, the expression of *HY5-4* homolog was significantly induced by the red light, indicating it may play a key role in the plant’s response to red light.

## Discussion

In the present study, different photoperiods under W or RB light were applied to examine growth and GS accumulation in Chinese kale sprouts. RB light caused the sprouts to achieve greater plant height and more dry matter than W light. Changes in photoperiodicity did not affect the rhythmic reduction of total GS content in the growing sprouts; however, RB light at different ratios in the 16-h light/8-h dark regimen affected the biosynthesis and degradation of GSs in 6-day-old sprouts. RNA-seq was applied to determine the differential accumulation of GSs under RB light.

### Accumulation of GSs in Sprouts Is Dominated by Catabolic Pathway

It is a common practice to up-regulate the expression level of synthetic genes to increase the content of specific secondary metabolites. Four types of genes are directly related to the final GS content in the sprouts: (1) side-chain extension genes *BCAT4*, *IPDMH*, *MAM 1*, and *MAM 2*; (2) core structure biosynthetic genes, e.g., *CYP79F1* and *CYP83A1*; (3) secondary modification genes, e.g., *FMOGS-OX* and *AOP2*; and (4) GS decomposition genes (myrosinase), e.g., *TGG*, *PEN2*, and *PYK10* (Figure 5).

In the present study, the GS content was lower under red light than under blue light, whereas expression of GS biosynthetic gene homologs (*BCAT4*, *MAM*, *CYP79F1*, and *CYP8A1*, etc.) showed the opposite trend. To our surprise, up-regulation of GS biosynthetic gene homologs did not result in higher accumulation of GSs under red light. The reasons for reduced GS content under red light could be related to the multiple sources of GSs and vigorous catabolism in the sprouts. Most GSs in sprouts are stored in seeds, which is gradually degraded to provide nutrients for other metabolic functions (Falk et al., 2007). During that process, myrosinase-like enzymes may play a key role in the degradation of GSs. Our RNA sequencing data showed that compared with HHB, expression of *TGG4* and *PYK10-1* homologs in HHR was significantly up-regulated, indicating that they may be critical for the reducing GSs under red light. Higher expression of GS catabolic gene homologs is accompanied by considerable GS decomposition, which ultimately leads to decreased GS content (Gao et al., 2014). One study reported that in the radish the myrosinase gene *TGG* was up-regulated by phototropic stimulation (Yamada et al., 2003).

Biosynthesis of GSs *de novo* would be another way to supply GSs in kale sprouts. However, although more transcripts of GS biosynthetic gene homologs including *BCAT4*, *MAM1*, *CYP83A1*, *SOT*, *AOP2*, and *FMOGS-OX* were detected, no increase in GS accumulation of sprouts was observed under red light. The increase in GS biosynthetic genes and the decreased GS content indicate that the degrading pathway of GSs is key to the change of sprouts GS content under different light conditions. However, the degradation of GSs in intact plant is in its infancy (Jeschke et al., 2019). The identification of atypical myrosinase PEN2/BGLU26 and PYK10/BGLU23 in the turnover of indolic GSs in intact plants (Clay et al., 2009; Nakano et al., 2017) may shed light on the clarification of GS degradation pathway. Taking into the abundant BGLU homologs identified in Chinese kale sprouts, the high expression of those BGLUs may be closely related to the response of GS pathway to different light treatments.

### Effect of Different Photoperiods on Appearance Quality

The appearance quality of vegetables is very important for sales, and in that regard, the phenotype is a significant factor. As a new type of vegetable, kale sprouts do not offer many choices with phenotype. Today, with advanced growing facilities, it is possible to provide better growing environments, allowing regulation of the various factors that make plants grow properly and improve vegetable quality. As an environmental factor that can affect plant morphogenesis, light has the advantages of being easily regulated, low cost, and safe (Jackson, 2009). Chinese kale has high nutritional value, easy seed acquisition, and low price, making it fit for mass production. In the present study, the morphological changes and characteristics of GS accumulation in Chinese kale sprouts at different times were studied under different photoperiods and light sources. The results demonstrated that longer illumination (16-h light/8-h dark) led to the formation of short, strong hypocotyls and expanded cotyledons with abundant dry matter; dark condition (0-h light/24-h dark) was better for vertical growth of the hypocotyls and undeveloped yellow cotyledons. This finding is consistent with the result that short-day conditions are especially appropriate for elongated hypocotyls (Niwa et al., 2009). It has been reported that PIF4 and PIF5 participated in regulating the photoperiodic elongation of hypocotyls: short photoperiods are essential for triggering the expression of *PIF4* and *PIF5* (Niwa et al., 2009). Further, to exclude the involvement of other constituents with W light, RB light was used at the ratio of 2:8, which mimicked the proportion of red and blue light in W light treatment. The result showed that under W light condition, the sprout height was less than that with RB light treatment, indicating that other constituents in W light may play a negative role in sprouts’ growth.

The photoperiod changes the appearance of kale sprouts mainly because the difference in light and dark regimes affects the length of the hypocotyl and width of the cotyledon. In the present study, we demonstrated that in addition to the photoperiod, changing the RB light ratio can affect the sprouts’ appearance. The sprouts were cultured at different RB light ratios, and the results showed that the sprouts’ height increased with a higher red light ratio, and the width decreased accordingly; an exception was with individual treatment with blue light. PHYs are responsible for recognizing red light. After treatment with red light, the expression of four *PHY* gene homologs in the sprouts was all up-regulated, and they released downstream genes controlled by the negative regulators *PIF*s, followed by the activation of the transcriptional factors *HY5*. Among the five HY5 members detected, higher expression of *HY5-4* homolog would be important for regulation of hypocotyl elongation. However, no *CRY* homolog transcripts were found in the two libraries analyzed. Further research should examine the regulation mechanism of sprout growth under blue light.

### Effect of Photoperiod With W or RB light on GS Accumulation

Glucosinolates are secondary metabolites present mainly in cruciferous plants. It has been reported that long illumination could promote GS accumulation in watercress and *Arabidopsis* (Engelen-Eigles et al., 2006; Huseby et al., 2013). However, our results do not concur with those earlier findings: we found that in Chinese kale sprouts, changing the photoperiod had no significant effect on GS content. This discrepancy could be due to varying GS profiles in different plant species, as well as the type of light sources used.

In addition, our results showed that the blue light application had a significant positive effect on GS accumulation in Chinese kale. The highest GS content was observed in sprouts grown under blue light, and the GS level in 6-day-old sprouts under blue light was comparable to that of 3-day-old sprouts under W light. This GS enhancement with blue light treatment has also been reported in *Arabidopsis* (Mewis et al., 2005). However, reduced GSs under blue light have also been found (Qian et al., 2016). Considering the different light sources used in various studies, it is possible that different results may be obtained with blue light as wide ranges of blue light wavelength (380–500 nm) exist.

## Conclusion

Our results showed that although changing the photoperiod had little effect on GS accumulation in the sprouts, it did exert a major influence on the appearance; it provided support for shaping the phenotype. RB light had a positive impact on the sprouts’ growth, with greater plant height and more dry matter. The lower accumulation of GSs and more transcripts of GS biosynthetic and degradation genes under red (versus blue) light leads us to conclude that the degrading pathway of GSs does exist in living sprouts and positively responds to the red light treatment. Identification of genes responsible for the degradation of GSs in intact plants is critical to understand the GS metabolism in growing plants and their response to environmental factors.

## Data Availability Statement

The datasets presented in this study can be found in online repositories. The names of the repository/repositories and accession number(s) can be found below: BioProject: PRJNA649862 2 Biosamples 6 SRAS: HHB (SRR12358308, SRR12474730, and SRR12474729) and HHR (SRR12358307, SRR12474732, and SRR12474731).

## Author Contributions

GW-P, RG, and XC designed the research. RG, JC, ZC, ZL, and YZ performed the research and wrote the manuscript. RG, JC, and ZC analyzed the data. All authors have read and approved the manuscript for publication.

## Conflict of Interest

The authors declare that the research was conducted in the absence of any commercial or financial relationships that could be construed as a potential conflict of interest.

## Footnotes

1 http://plants.ensembl.org/Brassica_oleracea/Info/Index

2 http://deweylab.biostat.wisc.edu/rsem/rsem-calculate-expression.html

3 http://www.integle.com/static/indraw

4 https://github.com/CJ-Chen/TBtools

## Supplementary Material

Supplementary Table 1 The individual GS content in sprouts at different stages under different photoperiodic conditions.

Supplementary Table 2 The individual GS content including four types of aliphatic GS (GIB, PRO, GNA, and GER) and indolic GS (4-OH GBS, GBS, 4-OM GBS, NGBS), respectively.

Supplementary Table 3 Genes identified in GS metabolism and light response pathway were listed.
